# Serum neurotransmitter analysis of motor and non-motor symptoms in Parkinson’s patients

**DOI:** 10.3389/fnagi.2024.1423120

**Published:** 2024-11-25

**Authors:** Yichun Fan, Wenping Yang, Weilan Wu, Xinjing Wang, Yuxin Lin, Linlin Wu, Jun Wang, Fei Huan, Haixia Ding, Rong Gao

**Affiliations:** ^1^Center for Global Health, School of Public Health, Nanjing Medical University, Nanjing, China; ^2^Department of Hygienic Analysis and Detection, the Key Laboratory of Modern Toxicology of Ministry of Education, School of Public Health, Nanjing Medical University, Nanjing, China; ^3^The First Affiliated Hospital of Nanjing Medical University, Jiangsu Provincial People’s Hospital, Nanjing, China; ^4^Affiliated Wuxi Center for Disease Control and Prevention of Nanjing Medical University, Wuxi Center for Disease Control and Prevention, Wuxi, China; ^5^Department of Toxicology, the Key Laboratory of Modern Toxicology of Ministry of Education, School of Public Health, Nanjing Medical University, Nanjing, China

**Keywords:** Parkinson’s disease, motor and non-motor symptoms, UPLC-MS/MS, neurotransmitter metabolism, tryptophan

## Abstract

Clinical symptoms of Parkinson’s disease (PD) are classified into motor and non-motor symptoms. Mental disorders, especially depression, are one of the major non-motor manifestations of PD. However, the underlying mechanisms remain poorly understood. In the present study, 21 neurotransmitters associated with mental disorders were measured in serum samples from patients and controls using the ultra-high performance liquid chromatography–tandem mass spectrometry (UPLC-MS/MS) assay. Additionally, five clinical scales—the MDS Unified Parkinson’s Disease Rating Scale (UPDRS), the Non-Motor Symptoms Scale (NMSS), the Mini-Mental State Examination (MMSE), the Hamilton Anxiety Scale (HAMA), and the Hamilton Depression Scale (HAMD)—were used to evaluate the severity of both motor and non-motor symptoms in PD patients. Analysis of neurotransmitter metabolism revealed significant changes in the tryptophan (Trp) metabolic pathway in PD patients. Specifically, levels of Trp, kynurenine (KYN), kynurenic acid (KA), nicotinamide (NAM), and 5-methoxyltryptamine (MeOTA) were substantially decreased. Additionally, three other excitation/inhibiting amino acids—glutamic acid (Glu), 4-aminobutyric acid (GABA), and aspartic acid (Asp)—also declined. Moreover, neurotransmitter conversion ratios, such as KA/KYN, nicotinamide/niacin (NAM/NA), 5-hydroxytryptophan/tryptophan (5-HTP/Trp), and quinolinic acid/kynurenic acid (QA/KA), provided more dynamic insights into disrupted neurotransmitter metabolism. Correlation analyses between scale scores and neurotransmitter levels showed that concentrations of xanthurenic acid (XA) and the turnover rate of 3-hydroxykynurenine (3-HK) were negatively correlated with UPDRS scores, while 5-hydroxytryptamine (5-HT) and GABA levels were negatively correlated with non-motor symptoms in PD patients. In summary, this study elucidates, for the first time, the potential association and dynamics between altered neurotransmitter metabolism and the etiology of PD in terms of motor and non-motor functions. These findings offer novel biomarkers and therapeutic targets for the treatment of PD.

## 1 Introduction

Parkinson’s disease (PD) is the second most prevalent neurodegenerative disorder worldwide, following Alzheimer’s disease, and it is a multifactorial condition with largely unknown etiology (Cacabelos, 2017; Frisardi et al., 2016). Currently, PD imposes a significant burden on society, affecting more than 1% of the population over the age of 60 years (Ó Breasail et al., 2022; Shao et al., 2021). A report from 2015 in Tianjin, China, indicated that the average annual cost of PD per patient had increased to $3,225.94, with direct medical expenses amounting to $1,737.93 (Li et al., 2019).

PD is characterized by a wide range of symptoms, encompassing both motor symptoms (e.g., bradykinesia, muscle tonus, postural balance disorders, and resting tremor) and non-motor symptoms (e.g., olfactory disturbances, sleep disturbances, urinary and fecal dysfunction, and depression; Armstrong and Okun, 2020; Zhang et al., 2022; Dong et al., 2023). Notably, depression is one of the most prominent non-motor manifestations and a common neuropsychiatric comorbidity in PD (Jiang et al., 2023). Approximately 40–50% of patients experience depression, with 17% of them suffering from major depressive disorder (Stocchi et al., 2023). Depression can exacerbate dyskinesia, impair cognitive function, and severely impact patients’ quality of life (Pontone et al., 2016). Notably, non-motor symptoms may precede the onset of motor symptoms, highlighting its value for early diagnosis and intervention.

Several studies have shown that numerous active metabolites of the tryptophan (Trp) metabolic pathway are involved in the pathogenesis of PD (Behl et al., 2021; Zhang et al., 2021; Lovelace et al., 2017; Lim et al., 2017). However, most of this research has focused on specific key neurotransmitters and animal models, with limited data available from population-based studies..

For the Trp metabolic pathway, approximately 95% of the Trp is converted into KYN, and the downstream metabolites and KYN act as modulators of the immune response and are associated with neurotoxicity and neuroinflammation in PD (Fathi et al., 2022). Even under healthy conditions, 3-hydroxykynurenine (3-HK) and 3-hydroxyanthranilic acid (3-HAA), the two key downstream metabolites of KYN, can lead to the production of large amounts of free radicals, cause oxidative stress and mitochondrial damage, and finalize central nervous system (CNS) disorders (Qin et al., 2022). Notably, the aberrant 3-HK increase facilitates downstream levels of quinolinic acid (QA), a toxic metabolite that activates the n-methyl-d-aspartate (NMDA) receptor, leading to excitotoxicity and accelerated inflammatory responses in a positive-loop property. Reciprocally, kynurenic acid (KA), another metabolite of KYN, serves as an antagonist of NMDA receptors and exhibits neuroprotective effects, attenuating PD symptoms in both *in vivo* and *in vitro* studies (Mor et al., 2021; Zhang et al., 2019).

The 5-hydroxytryptamine-Trp metabolic pathway is recently reported to be associated with the pathogenesis of PD, which displays significant alterations in the 5-HT-ergic system, such as reduced levels of 5-hydroxyindole acetic acid (5-HIAA) and reduced expression of the rate-limiting enzyme of 5-HT biosynthesis, tryptophan hydroxylase (TPH; Jing et al., 2023; Zárate et al., 2022; Pasquini et al., 2018). Studies have shown that 5-HT transporter (SERT) availability is reduced in the caudate-putamen region in human carriers of mutations associated with genetic PD, and the alteration precedes dopaminergic lesions and the onset of motor symptoms, suggesting that 5-HT ergic dysfunction may serve as an early warning of PD (Wile et al., 2017; Wilson et al., 2019).

In addition to the Trp metabolic pathway, some other inhibitory or excitatory neurotransmitters also exert critical roles in the development of PD. It was recently found that aberrant expression of 4-aminobutyric acid (GABA) transporters may lead to olfactory dysfunction in a PD mouse model, an effect that was significantly improved after the treatment of GABA reuptake agents (Liu et al., 2023). Meanwhile, previous magnetic resonance spectroscopy (MRS) studies in PD patients revealed an increased GABA/Glu ratio in the substantia nigra (Öz et al., 2006). The precursor substance of GABA, glutamate (Glu), mediates the excitatory signals. Accumulation of glutamate beyond physiological limits at the synapse is toxic and triggers apoptosis due to Ca^2+^ overload upon overstimulation of glutamate receptors in PD (Iovino et al., 2020). In addition, as an active ingredient in the treatment of muscle-related movement disorders, the excitatory neurotransmitter aspartate (Asp) and the inhibitory neurotransmitter glycine (Gly) have been found to be homeostatically disrupted in the plasma of PD patients (Iwasaki et al., 1992).

**Figure 1:**
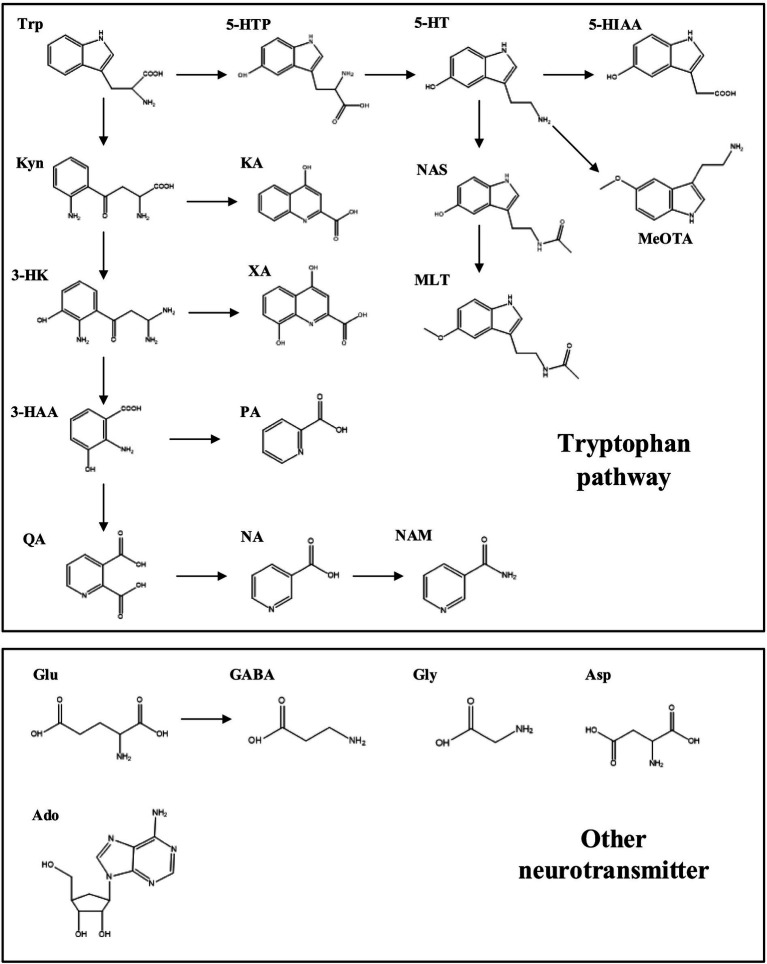
Neurotransmitter metabolic pathways of tryptophan and excitatory/inhibitory amino acids. Neurotransmitters and metabolites: Trp, tryptophan; KYN, kynurenine; 3-HK, 3-hydroxykynurenine; 3-HAA, 3-hydroxyanthranilic acid; KA, kynurenic acid; XA, xanthurenic acid; QA, quinolinic acid; PA, picolinic acid; NA, niacin; NAM, nicotinamide; 5-HTP, 5-hydroxytryptophan; 5-HT, 5-hydroxytryptamine; 5-HIAA, 5-hydroxyindole acetic acid; NAS, N-acetyl-5-hydroxytryptamine; MLT, melatonin; MeOTA, 5-methoxyltryptamine; Glu, glutamic acid; GABA, 4-aminobutyric acid; Gly, Glycine; Asp, aspartic acid; Ado, adenosine.

However, there are few studies on the correlation between non-motor symptoms and disturbed neurotransmitter metabolism in serum in patients with PD. Thereby, the ultra-high performance liquid chromatography–tandem mass spectrometry (UHPLC–MS/MS) assay was exploited in the present study to investigate the serum amino acid metabolism, including the Trp pathway and the excitatory/inhibitory amino acid metabolic pathway in PD patients (Figure 1). Meanwhile, the correlation between the levels of the neurotransmitters and the PD-related symptom scale scores was assessed, which may provide novel insights into the clues and rationale for diagnosing and treating clinical PD.

## 2 Materials and methods

### 2.1 Patient and control recruitment

This study was conducted in collaboration with Jiangsu Provincial People’s Hospital and approved by the Ethics Committee of Nanjing Medical University (Ethics No.2022-SR-372). All participants signed an informed consent form. Middle-aged and elderly patients (aged 50 years and above) who clinically presented to the hospital with complaints of Parkinson’s disease were selected for the study. Patients with Parkinson’s disease were diagnosed and assessed according to the MDS Unified-Parkinson Disease Rating Scale (UPDRS), the Non-Motor Symptoms Scale (NMSS), the Mini-Mental State Examination (MMSE), the Hamilton Anxiety Scale (HAMA), and the Hamilton Depression Scale (HAMD). Meanwhile, baseline demographic information on individuals was collected from the patient’s hospital registry at the time of admission. The control group consisted of inpatients from other departments whose gender and some demographic information matched those in the medical record group, and they were assessed using the scale and by professional clinicians to ensure that they did not have Parkinson’s disease. In addition, the exclusion criteria for all individuals included (1) non-secondary or atypical Parkinson’s disease, (2) family history of Parkinson’s disease, and (3) inability to complete clinical assessments.

### 2.2 Sample collection

Fasting blood was collected and centrifuged, after which the supernatant was dispensed into 2 ml centrifuge tubes and wrapped in plastic sealing film to prevent leakage. The samples were immediately transferred to a −80°C refrigerator for storage.

### 2.3 UHPLC–MS/MS neurotransmitter analysis

Two different UHPLC–MS/MS methods were applied to detect neurotransmitters in serum samples. Method I detected tryptophan and its metabolites (except MLT and NAS), and method II detected MLT, NAS, and the excitatory/inhibitory amino acids and their metabolites. Methods I and II use different internal standard (IS) substances.

The instrument setup consists of the Ulimate 3000 Ultra High-Performance Liquid Chromatograph (UHPLC) of Thermo Fisher Scientific (Waltham, America) and the Q Exactive four-stage rod orbit trap high-resolution mass spectrometer (Q Exactive) of Thermo Fisher Scientific in tandem. Xcalibur software was used for instrument control, as well as for data acquisition and analysis.

#### 2.3.1 Sample preparation

In a 1.5 ml centrifuge tube, 100 μl serum, 50 μl ISs, and 400 μl methanol were accurately added and vortexed for 30 s. The mixture was centrifuged in a cryogenic high-speed centrifuge at 4°C and 12,000 rpm for 10 min. All supernatants were transferred to a new 1.5 ml centrifuge tube and volatilized dry by placing them in a cryogenic vacuum centrifugal concentration system.

Method I: After evaporation, 100 μl of initial mobile phase (water:acetonitrile = 99:1, formic acid 0.1%) was added, vortexed for 1 min, and centrifuged at 12,000 rpm for 10 min at 4°C. Then, 40 μl of the supernatant was transferred to a brown glass-lined injection bottle, and the injection volume was 2 μl.

Method II: After evaporation, 50 μl of NaHCO_3_ (pH = 9, 0.2 M) and 50 μl of Dansyl chloride (2 mg/ml, dissolved in acetone) was added into the centrifuge tube, vortexed, and mixed for 1 min. The mixture reacted in a water bath at 60°C for 8 min and then centrifuged at high speed at 12,000 rpm for 10 min at 4°C. Then, 50 μl of the supernatant was taken in a brown injection vial with a glass-lined tube, and the injection volume was 2 μl.

#### 2.3.2 UHPLC–MS/MS analysis

In all the methods using UHPLC–MS/MS in this study, mass spectra were performed using a heated electrospray ionization source (HESI) for analyte ionization and multiple reaction monitoring (MRM) modes for quantitative analysis.

Method I: An Acquity UPLC HSS T3 column (100 × 2.1 mm, 1.8 μm) was used. The column temperature was 40°C. The mobile phase composition was as follows: mobile phase A contained water containing 0.1% formic acid, and mobile phase B contained acetonitrile containing 0.1% formic acid. The gradient elution procedure was as follows: 0–0.5 min, 1%B; 0.5–2 min, 1–70% B; 2–4 min, 70–95% B; 4-5 min, 95%B; 5–5.1 min, 95–1% B; and 5.1-7 min, 1%B. The total run time and flow rate were 7 min and 0.25 min/ml, respectively. The mass spectrometry parameters were set as follows: the electrospray voltage was set at 30 kV, the capillary temperature was 350°C, heater temperature was 500°C, the sheath gas flow rate was 55 arb, the auxiliary gas flow rate was 55 arb, and the purge gas flow rate was 35 arb.

Method II: An Acquity UPLC BEH C_18_ column (100 mm × 2.1 mm, 1.7 μm) was used. The column temperature was 35°C. The mobile phase composition was as follows: mobile phase A contained water containing 0.1% formic acid, and mobile phase B contained acetonitrile containing 0.1% formic acid. The gradient elution procedure was as follows: 0–1 min, 10% B; 1–4 min, 10-60%B; 4–6 min, 60%B; 6–15 min, 60–95% B; 15–17 min, 95% B; 17–17.1 min, 95-10%B; and 17.1–20 min, 10%B. The total run time and flow rate were 20 min and 0.25 min/ml, respectively. The parameter settings for the mass spectrometry were specified as follows. The electrospray voltage was 30 kV, the capillary temperature was 350°C, the heater temperature was 280°C, the sheath gas flow rate was 46 arb, the auxiliary flow rate was 10 arb, and the purge gas flow rate was 3 arb.

### 2.4 Statistical analysis

SPSS 26.0 software was used for the statistics and analysis of the experimental data, and GraphPad Prism 8.0 was used to draw statistical graphs. For the comparison of dichotomous data between the two groups, the chi-squared test was used for those that met the four-cell test criteria, and Fisher’s exact test was used for those that did not meet this criteria. For quantitative data between the two groups, the distribution was first evaluated using the normality test. If the data were normally distributed, the two groups were represented by Mean ± SEM; if the data were not normally distributed, they were represented by the median. A non-parametric test is used to compare the data with a non-normal distribution. For data with normal distribution, if the chi-squared test yields a *p*-value >0.05, Student’s t-test was used for comparison; for data with uneven variance (*p* < 0.05), Welch’s t-test was used for comparison. For data conforming to a normal distribution, the Pearson correlation coefficient was used to analyze the data; otherwise, the Spearman correlation coefficient was used to analyze the correlation, and a p-value of <0.05 was considered statistically significant.

MetaboAnalyst 4.0 was used for multivariate statistical analysis of principal component analysis (PCA), orthogonal partial least squares discriminant analysis (OPLS-DA), and sparse partial least squares discriminant analysis (SPLS-DA) models.

## 3 Results

### 3.1 Demographics and clinical information

Detailed clinical data are presented in Supplementary Table S1. A total of 27 Parkinson’s patients and 19 participants from the control population who met the criteria were included in this study. Demographic data such as age, gender, and body mass index (BMI) were analyzed. As shown in Table 1, the age distribution, sex ratio, and clinical indicators were similar between the two populations (all *p* > 0.05).

**Table 1:** Basic information on Parkinson’s patients and controls.

Variables	Controls	Parkinson’s disease patients	*p*-value
Male	19 (10)	27 (10)	0.371
Age (years)	71.2 ± 3.8	67.1 ± 1.7	0.347
Height (cm)	166.9 ± 1.7	162.2 ± 1.6	0.065
Weight (kg)	67.0	62.0	0.160
BMI	23.2	23.4	0.691

### 3.2 Alterations of neurotransmitters and the corresponding metabolites in the serum of Parkinson’s patients

#### 3.2.1 Multivariate statistical analysis

**Figure 2:**
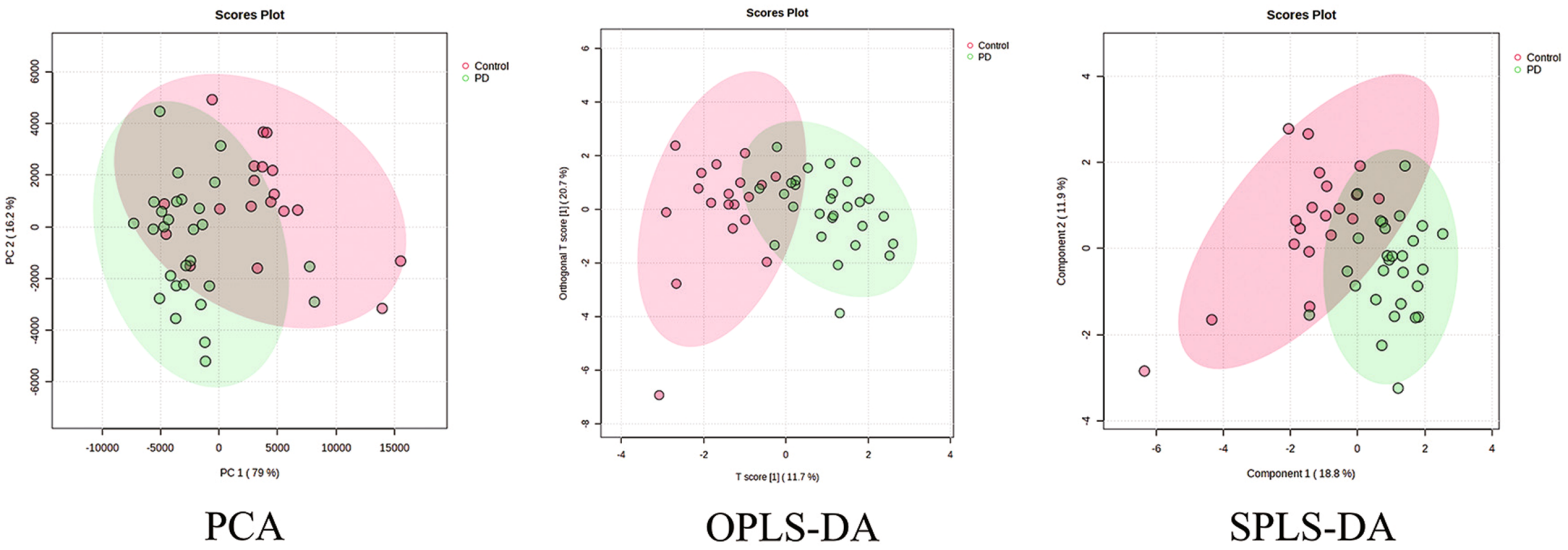
PCA, OPLS-DA, and SPLS-DA plots of neurotransmitter metabolic changes in the serum of the PD and control groups.

Serum samples from the PD patients and control population were collected, and the neurotransmitter levels were detected using the UHPLC–MS/MS assay. It showed that there was a significant difference in the neurotransmitter levels between the two groups (Table 2). Based on the results, the multivariate statistical analysis model was performed to analyze the overall changes (Figure 2). The PCA can characterize the distribution of data through data dimensionality reduction. The OPLS-DA can weaken intragroup differences and highlight intergroup differences. The SPLS-DA can classify samples by selecting the most discriminatory features in the data. The PCA, OPLS-DA, and SPLS-DA models were used to investigate the overall changes in neurotransmitter metabolism. The PCA model showed a slight separation between the Parkinson’s patient group and the control group, whereas a more pronounced separation between the PD group and the control group was found by both the OPLS-DA and SPLS-DA models, suggesting the disturbed metabolism of neurotransmitters in the serum of Parkinson’s patients.

**Table 2:** Serum levels of neurotransmitters and the corresponding metabolites in control and PD groups.

Compounds	Control	PD	Pathway
Trp	8080.55	7168.52**	Tryptophan pathway
KYN	820.91	390.88****	
3-HK	15.85	12.67	
KA	19.27	14.93**	
3-HAA	6.98	5.96	
XA	103.90	95.14	
QA	170.47	168.95	
PA	11.42	12.91	
NA	4.08	3.51	
NAM	72.27	20.85****	
5-HTP	8.57	8.32	
5-HT	76.39	92.04	
5-HIAA	78.75	137.06	
NAS	0.54	0.48	
MLT	0.26	0.16	
MeOTA	1.45	1.17*	
Glu	14504.58	7330.04***	Other neurotransmitter
GABA	32.73	28.27*	
Gly	12961.44	13195.38	
Asp	2815.71	1769.34****	
Ado	3.50	2.65	

ps: All data are expressed as medians, and statistically different substances are shaded in gray. Substances were tested using non-parametric tests to determine significant differences between PD patients and controls. *p < 0.05, **p < 0.01, ***p < 0.001, and ****p < 0.0001 vs. the control group.

#### 3.2.2 Non-parametric tests and independent samples t-tests

After identifying the significantly altered neurotransmitters in the serum of Parkinson’s patients, we proceeded to investigate the target neurotransmitter metabolism in the serum.

**Figure 3:**
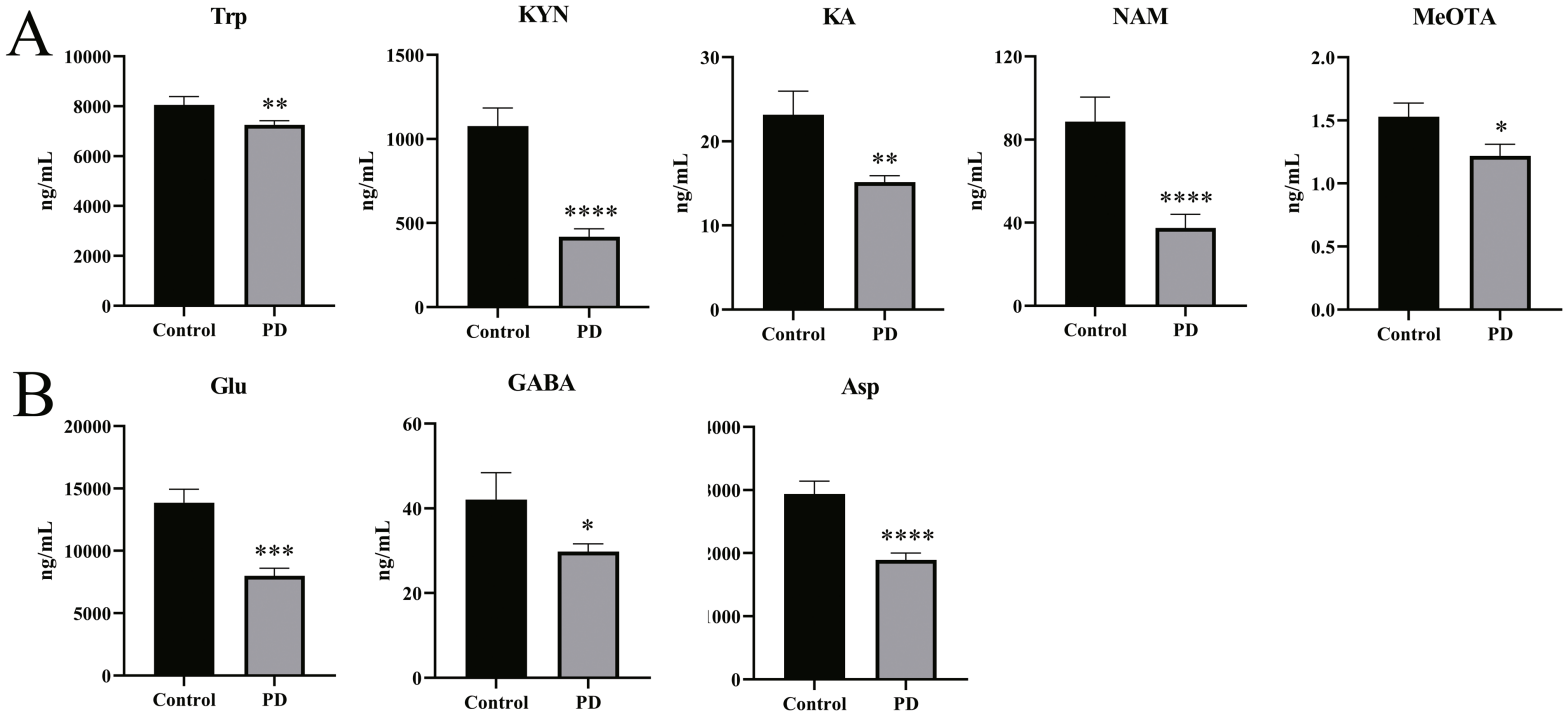
Differences in neurotransmitter levels in serum samples from Parkinson’s patients and controls. **(A)** Differences in levels of partial Trp pathway metabolites in serum samples from Parkinson’s patients and controls. **(B)** Differences in levels of Glu, GABA, and Asp in serum samples from Parkinson’s patients and controls. All non-parametric tests were used. *p < 0.05, **p < 0.01, ***p < 0.001, and ****p < 0.0001 vs. the control group.

Table 2 and Figure 3A show substantial differences in the neurotransmitter levels in Trp metabolism in the Parkinson’s patient group compared to the control group. Significant changes in the levels of tryptophan and some of the active metabolites in its metabolic pathway were observed in detail. Serum levels of Trp were significantly decreased in Parkinson’s patients compared to controls (*p* = 0.006), with a commensurate decrease in the levels of one of its key downstream active metabolites, KYN (*p* < 0.0001). KA, an important neuroprotective substance on the Trp-KYN pathway, significantly decreased in the serum of Parkinson’s patients (*p* = 0.008). Meanwhile, other neurotransmitters in the Trp-KYN metabolic pathway, including 3-HAA, 3-HK, and QA, were not statistically different between the Parkinson’s patient group and the control group. NAM, the terminal substance of the Trp-KYN metabolic pathway, exhibited a decreased trend in the serum of Parkinson’s patients (p < 0.0001). Meanwhile, the level of the terminal substance MeOTA in the serum of Parkinson’s patients was significantly lower than that of controls (*p* = 0.015) in the Trp-5-HT metabolic pathway.

In addition to the tryptophan metabolic pathway, a number of neurotransmitters associated with excitatory/inhibitory amino acids and the corresponding metabolites were detected. As shown in Figure 3B, the excitatory neurotransmitters Glu (*p* < 0.001) and Asp (*p* < 0.0001) were significantly decreased in the serum of Parkinson’s patients compared to the control group. Similarly, GABA, a metabolite of Glu, also displayed a significant decrease in the serum of Parkinson’s patients (*p* = 0.022). These results suggest a disturbance of the neurotransmitter homeostasis associated with the Trp and the excitatory/inhibitory amino acid metabolic pathways in Parkinson’s patients.

### 3.3 Altered neurotransmitter dynamics in the serum of Parkinson’s patients

The dynamic metabolism of the neurotransmitter reflects the direction of neurotransmitter conversion, which serves as a critical index for disease assessment (Kim et al., 2024).

**Figure 4:**
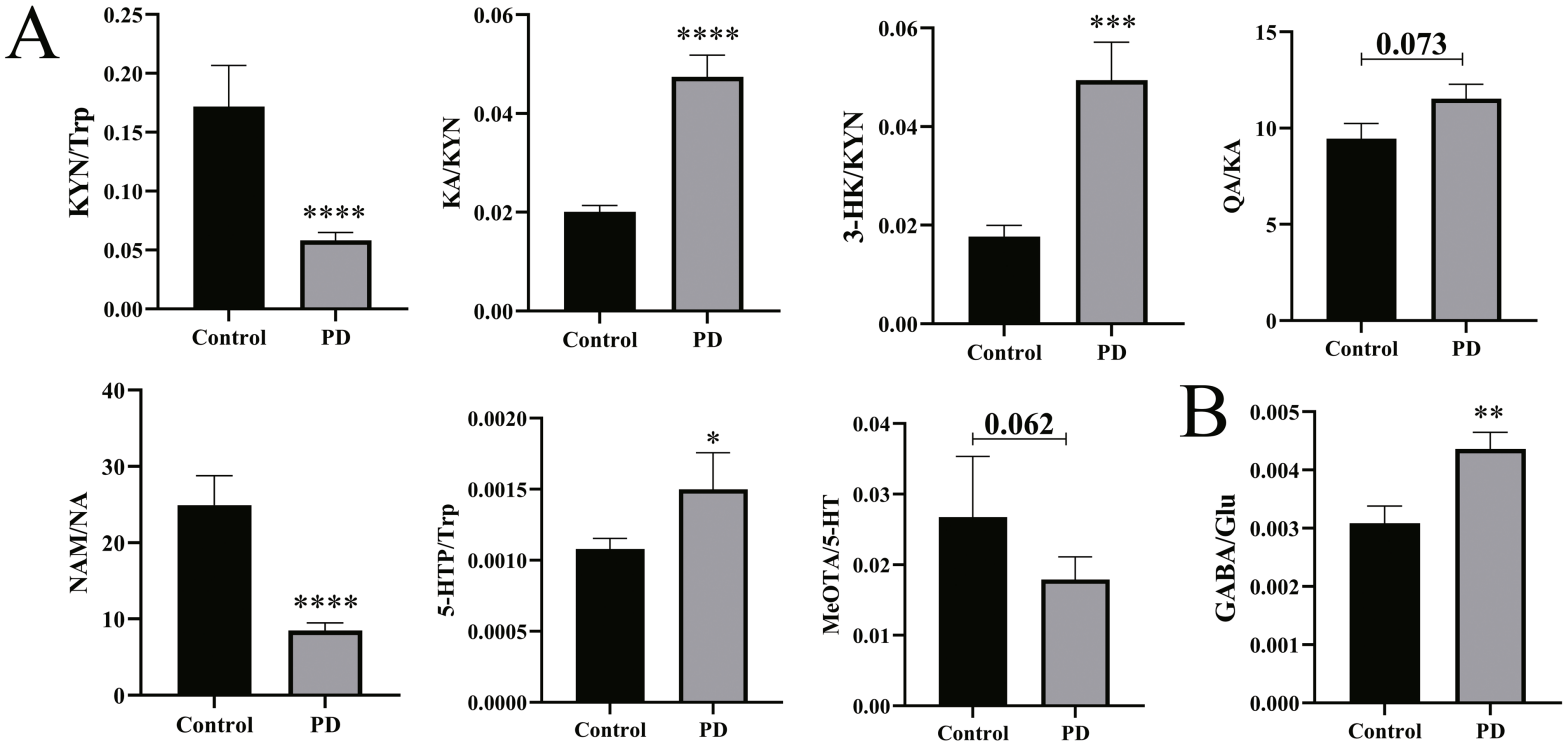
Differences in neurotransmitter synthesis and metabolism in serum between Parkinson’s patients and controls. **(A)** Difference in neurotransmitter dynamic metabolism in the Trp pathway. **(B)** Differences in the GABA/Glu ratio. Non-parametric tests were used. *p < 0.05, **p < 0.01, ***p < 0.001, and ****p < 0.0001 vs. the control group.

Therefore, the alteration assessed by the ratio of the critical neurotransmitters was investigated. As depicted in Figure 4, the GABA/Glu ratio was significantly increased (*p* = 0.005), indicating that the metabolic rate of Glu is increased in PD patients and that the pathways involved in the production of GABA are aberrantly activated. Meanwhile, for the Trp metabolic pathway, the KA/KYN (*p* < 0.0001), 3-HK/KYN (*p* < 0.001), and 5-HTP/Trp (*p* = 0.025) ratios were significantly increased. Reciprocally, the NAM/NA (*p* < 0.0001) and KYN/Trp (*p* < 0.0001) ratios were significantly decreased. In addition, the QA/KA (*p* = 0.073) ratio displayed an increased tendency, and the MeOTA/5-HT (*p* = 0.062) displayed a decreased tendency. These results, taken together, suggest the disturbed Trp metabolism and the aberrant metabolic conversion of the neurotransmitters in PD patients.

### 3.4 Correlation between behavioral scale scores and neurotransmitters in Parkinson’s patients

After determining the differences in the neurotransmitter levels and the distinct dynamic metabolic properties of the aforementioned neurotransmitters, we then explored whether there were some potential links between the aberrant neurotransmitter metabolism and the behaviors of Parkinson’s patients and healthy individuals.

**Figure 5:**
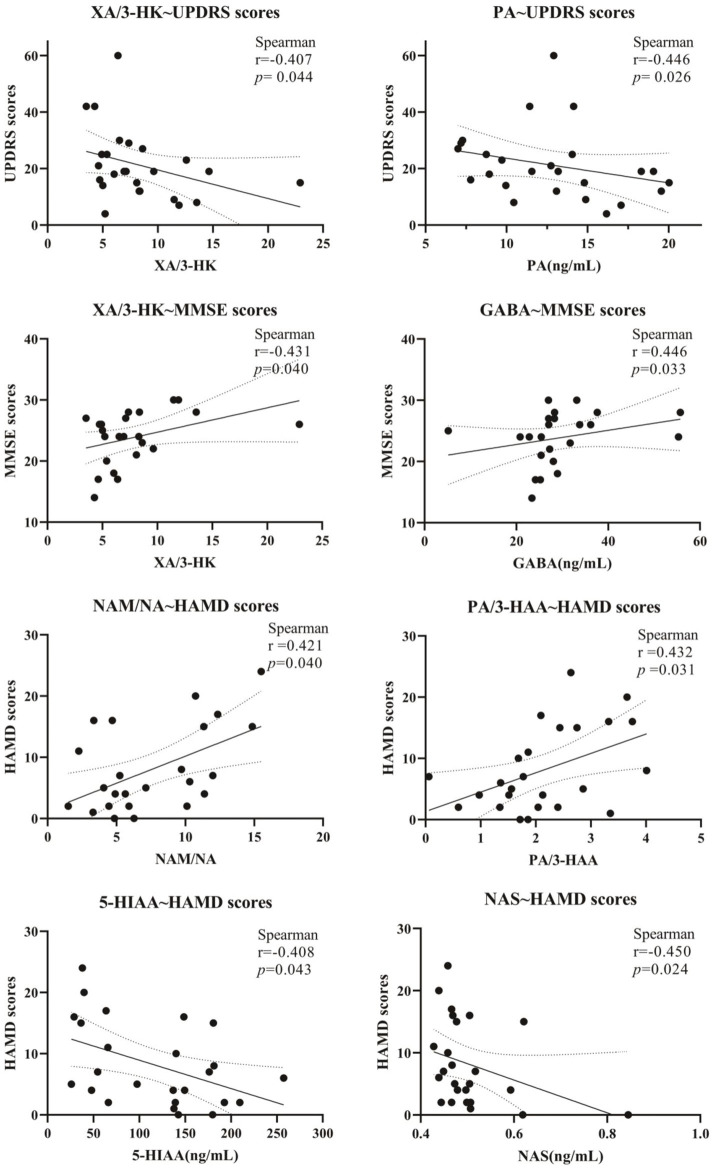
Correlation of Clinical Symptom Scale scores with neurotransmitters in Parkinson’s patients. The correlation between scale scores and serum metabolite levels is expressed as Spearman’s *r*-value.

**Figure 6:**
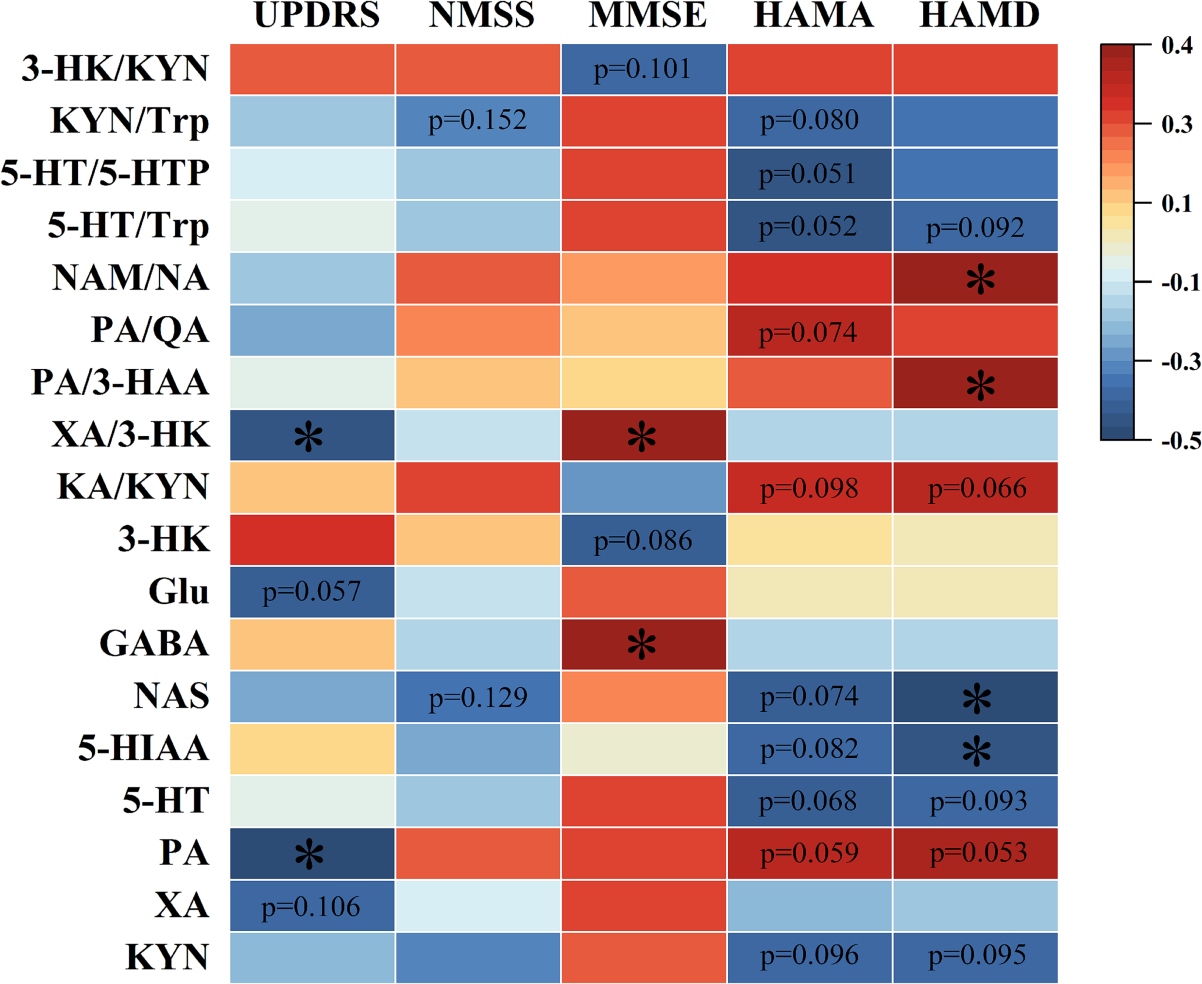
Spearman’s correlation heatmap of the correlation between neurotransmitters in Parkinson’s serum and clinical scale scores. The scale (right legend) indicates the degree of positive (red) or negative (blue) correlation, and asterisks indicate significance (*p < 0.05).

Five scales, including UPDRS, NMSS, MMSE, HAMA, and HAMD, were used for the behavior test. The results are shown in Figures 5, 6. It showed that the levels of Glu, XA, and PA and the ratio of XA/3-HK were negatively correlated with the UPDRS scores; NMSS scores exhibited a negative correlation with the NAS level and the KYN/Trp ratio. In addition, the level of GABA and the XA/3-HK ratio displayed a positive association with MMSE scores, whereas the level of 3-HK and the 3-HK/KYN ratio were negatively associated with MMSE scores. Notably, Trp and its metabolites are significantly associated with HAMA scale scores. It showed that HAMA scale scores negatively correlated with the levels of KYN, 5-HT, 5-HIAA, NAS, 5-HT/5-HTP, 5-HT/Trp, and KYN/Trp ratios. Concomitantly, the PA level, KA/KYN, and PA/QA ratios were positively correlated to some extent with HAMA scores. For the depression test, the HAMD scale scores and neurotransmitter correlations were similar to those of the HAMA scale. Figures 5, 6 indicated that the levels of KYN, 5-HT, 5-HIAA, and NAS of the Trp metabolic pathway and the 5-HT/Trp ratio were negatively associated with HAMD scores. Reciprocally, the PA levels, KA/KYN, PA/3-HAA, and NAM/NA ratios were positively associated with HAMD scale scores. However, the 5-HT/Trp and KA/KYN ratios and the 5-HT, PA, and KYN levels displayed no statistical significance. These results suggest that the abnormal neurotransmitter levels and the disorder of conversion metabolism in PD patients may have potential applications as biomarkers to assist in the diagnosis of PD.

## 4 Discussion

The clinical research on the pathophysiology of PD has made great progress in recent decades. PD, one of the major neurodegenerative diseases, has a complex etiology and possesses not only clinical heterogeneity but also complex motor and non-motor symptoms (Cacabelos, 2017; Armstrong and Okun, 2020; Yuan et al., 2013). The absence of dopaminergic neurons in the striatum and substantia nigra is a key event of Parkinson’s disease, pathologically manifested as the aggregation of α-synaptic proteins in the substantia nigra with a number of other proteins to form a toxic protein known as Lewy bodies. The deposition of this toxic protein stimulates the activation of microglia, which releases inflammatory factors and exacerbates neuronal damage (Sarkar et al., 2016). Notably, in the early stages of PD, the non-motor symptoms (NMS) often precede motor symptoms (Morimoto et al., 2023). NMS is present not only in the prodromal stage but also in all stages of the dyskinesia of PD and even in the final palliative stage (Titova and Chaudhuri, 2018). Notably, the presence of NMS not only reduces the quality of life of the patients but also aggravates their motor symptoms, such as gait disturbances (Avanzino et al., 2018). Most of the occurrences of NMS have been shown to be associated with neurotransmitter metabolic disorders (Gu et al., 2024; Ahmad et al., 2023; Samizadeh et al., 2023). Therefore, we assumed aberrant neurotransmitter metabolism may directly or indirectly reflect the alterations of PD in the central nervous system. In the current study, the serum levels of the metabolites from the Trp metabolic pathways and other key neurotransmitters (Glu, GABA, Gly, Asp, and Ado) were investigated. Moreover, the correlations between the aberrant alteration of neurotransmitters and the clinical behaviors were addressed in PD patients, which may help in the diagnosis and treatment in the early stage of PD.

In the present study, the serum levels of neurotransmitters in PD patients differed from those in the serum of the control population. Notably, abnormal levels of the active metabolites associated with the Trp metabolic pathway have gained more attention for their potential roles as novel biomarkers for PD. Sorgdrager et al. found lower levels of Trp in the peripheral blood of patients with PD compared to controls, which is partially in accordance with our results in the current study (Sorgdrager et al., 2019). Moreover, our studies expanded the research into the downstream metabolic pathways of the Trp and found that KYN, one key metabolite of Trp, was significantly decreased. It supported the conclusion made by Heilman et al. that KYN levels decreased in cerebrospinal fluid and plasma in PD patients. However, it was in contrast to the results of Bai et al. observed in the urine of Parkinson’s patients. The reasons may be complex, but we suspect that different samples and stages in PD patients were involved, and more research pertaining to Trp metabolism is required in PD patients (Heilman et al., 2020; Bai et al., 2021).

KA is one of the important metabolites of KYN and has neuroprotective effects due to its ability to scavenge reactive oxygen species and extracellular glutamate (Platten et al., 2019). Reciprocally, QA, another metabolite of KYN, exhibited neurotoxicity and enhanced glutamatergic signaling, which is considered a potential biological mechanism for depression (Brown et al., 2023). As expected, the level of KA in the peripheral blood of PD patients in the current work was significantly decreased, accompanied by an increased QA/KA ratio, indicating the KP metabolism converted to a toxic pathway, in which the rate-limited enzymes, such as KATs, 3-HAO, IDO, and TDO may play pivotal roles. In addition, the 5-HT metabolic pathway, another key metabolic pathway of Trp (Zhu et al., 2020), is also impaired in PD patients. NAS, a precursor to melatonin in the Trp metabolic pathway, provides neuroprotection and prevents oxidative stress damage from H_2_O_2_ (Álvarez-Diduk et al., 2015). In this study, we found that there was a trend of decreasing NAS concentration in the serum of PD patients. MeOTA is a close metabolite of 5-HT and that the MeOTA/5-HT ratio decreases (Edwards and Zup, 2021). This evidence suggests that the protective metabolic pathway of 5-HT in PD patients was attenuated.

In addition to the Trp metabolic pathway, some other neurotransmitters key to psychiatric disorders were also altered in PD patients, including GABA, Glu, and Asp. Recently, it has been shown that α-synuclein interacts with microtubule β—iii to form a toxic complex, which may affect the release of neurotransmitters from interneurons such as GABA; in parallel, α-synuclein reduces Glu concentrations, and similar results were also found in paraquat and rotenone-induced models of PD (Lei et al., 2014; Mally et al., 1997). Dysregulation of the GABA pathway in Parkinson’s disease triggers neuronal hyperexcitability, leading to dyskinesia or bradykinesia. In a recent study, Ali et al. found that GABA treatment is able to, at least in part, prevent PD by modulating Ca^2+^−related ion channels to block excitotoxicity, oxidative stress, and mitochondrial dysfunction induced by elevated Ca^2+^ concentrations (Ali et al., 2024). Meanwhile, Lemos et al. found that GABA deficiency may lead to increased striatal discharge, resulting in motor retardation in PD patients (Lemos et al., 2016). Reciprocally, GABA agonist administration improved motor symptoms in Parkinson’s model rats (Hajj et al., 2015). For Glu, it is a precursor substance of GABA. Elevated levels of Glu in the medial substantia nigra/pallidum drive inhibition of thalamocortical feedback, which leads to motor dysfunction. However, there is no increase in levels of Glu in the medial substantia nigra/pallidum in PD mice when tested directly (Obeso et al., 2008; Gerlach et al., 1996). The Glu level was decreased in the current study, accompanied by GABA reduction. It may ascribe the GABA reduction to the decrease of Glu, which can be metabolized by glutamic acid decarboxylase (GAD), and further study will be performed on the expression of the GAD levels. As an important amino acid that regulates mitochondrial function, Asp can also be involved in the synthesis or metabolism of a number of substances that play a role in the development of neural tissue and neurotransmission (Holeček, 2023). For example, its brain-metabolized derivative, N-acetyl aspartate, is a potent inhibitor of protein aggregation, which may play a role in Parkinson’s disease (Warepam et al., 2021). The results of our study show that in PD patients, Asp levels in serum were significantly decreased, which is consistent with the study of Yuan et al. (2013). These pieces of evidence implied that changes in GABA, Glu, and Asp are closely related to PD development, which may serve as potential biomarkers, but the specific roles of these substances in Parkinson’s disease need to be further investigated.

A series of clinical checklists can be used to assess motor and non-motor symptoms and the life quality of PD patients, etc., through which more accurate quantitative studies can be conducted. The scales used in this study are UPDRS, NMSS, MMSE, HAMA, and HAMD. The UPDRS is the internationally recognized and most commonly used screening scale to comprehensively assess a patient’s motor function, the day-to-day impact of motor and non-motor symptoms of PD, and disease severity. In this study, we found that PD patients with increased levels of XA, accompanied by an enhanced XA/3-HK ratio, exhibited lower UPDRS scores and improved PD-like symptoms. It strengthened the evidence of Heilman et al., who found a significant decrease in 3-HK levels when using mass spectrometry for targeted metabolite assays in plasma of PD patients (Heilman et al., 2020). Meanwhile, it has been found that XA can interact with glutamate receptors and can show antipsychotic-like effects (Fazio et al., 2016), which was in line with the results of the present study.

Non-motor symptoms are very common and adversely impact patients’ quality of life. The NMSS, MMSE, HAMA, and HAMD used in this study are commonly used in clinical practice and can be used to assess the mental status, cognitive function, and depression or anxiety of PD patients. It was found that Trp and its related neuroactive substances of the 5-HT metabolic pathway were strongly associated with non-motor symptomatic processes in PD patients. It has been shown that depressive symptoms are the main non-motor symptom of PD, and patients treated with 5-HT reuptake agents showed a significant improvement in depression-like behaviors, which is in accordance with our previous findings that activation of 5-HT and 5-HIAA metabolic pathways negatively correlate with depression and anxiety symptom severity (Wang et al., 2022).

To the best of our knowledge, this study is the first to reveal metabolic changes in the Trp metabolic pathways and other key neurotransmitters (such as Glu, GABA, Gly, and Asp) in the peripheral blood of PD patients, along with their associations with motor and non-motor symptoms. However, there were still some limitations in the current study: first, the sample size of PD patients and controls was relatively small, and future studies should include a larger cohort. Second, this study assessed neurotransmitter levels only in peripheral blood, whereas cerebrospinal fluid measurements would provide more accurate insights. Finally, although we characterized neurotransmitter metabolism using ratios of altered neurotransmitter levels, further investigation of relevant rate-limiting enzymes is warranted.

## 5 Conclusion

In summary, the present study revealed an unrecognized disturbed neurotransmitter metabolism in PD patients, which is closely associated with both motor and non-motor symptom processes. Consequently, targeting neurotransmitter metabolism might provide novel insights into potential therapeutic strategies for preventing and treating PD.

## Data Availability

The original contributions presented in the study are included in the article/Supplementary material. Further inquiries can be directed to the corresponding authors.

## References

[ref1] Ahmad M. H. Rizvi M. A. Ali M. Mondal A. C. (2023). Neurobiology of depression in Parkinson's disease: insights into epidemiology, molecular mechanisms and treatment strategies. Ageing Res. Rev. 85:101840. doi: 10.1016/j.arr.2022.101840, PMID: 36603690

[ref2] Ali N. H. Alhamdan N. A. al-kuraishy H. M. al-Gareeb A. I. Elekhnawy E. Batiha G. E.-S. (2024). Irisin/PGC-1α/FNDC5 pathway in Parkinson's disease: truth under the throes. Naunyn Schmiedeberg's Arch. Pharmacol. 397, 1985–1995. doi: 10.1007/s00210-023-02726-9, PMID: 37819389

[ref3] Álvarez-Diduk R. Galano A. Tan D. X. Reiter R. J. (2015). N-Acetylserotonin and 6-Hydroxymelatonin against oxidative stress: implications for the overall protection exerted by melatonin. J. Phys. Chem. B 119, 8535–8543. doi: 10.1021/acs.jpcb.5b04920, PMID: 26079042

[ref4] Armstrong M. J. Okun M. S. (2020). Diagnosis and treatment of Parkinson disease: a review. JAMA 323, 548–560. doi: 10.1001/jama.2019.22360 32044947

[ref5] Avanzino L. Lagravinese G. Abbruzzese G. Pelosin E. (2018). Relationships between gait and emotion in Parkinson's disease: a narrative review. Gait Posture 65, 57–64. doi: 10.1016/j.gaitpost.2018.06.171, PMID: 30558947

[ref6] Bai J.-h. Zheng T.-l. Yong-peng Y. (2021). Urinary kynurenine as a biomarker for Parkinson's disease. Neurol. Sci. 42, 697–703. doi: 10.1007/s10072-020-04589-x, PMID: 32661882

[ref7] Behl T. Kaur I. Sehgal A. Singh S. Bhatia S. al-Harrasi A.. (2021). The footprint of kynurenine pathway in neurodegeneration: Janus-faced role in Parkinson's disorder and therapeutic implications. Int. J. Mol. Sci. 22:6737. doi: 10.3390/ijms22136737, PMID: 34201647 PMC8268239

[ref8] Brown S. J. Christofides K. Weissleder C. (2023). Sex- and suicide-specific alterations in the kynurenine pathway in the anterior cingulate cortex in major depression. Neuropsychopharmacology 49, 584–592. doi: 10.1038/s41386-023-01736-8 37735504 PMC10789861

[ref9] Cacabelos R. (2017). Parkinson's disease: from pathogenesis to pharmacogenomics. Int. J. Mol. Sci. 18:551. doi: 10.3390/ijms18030551, PMID: 28273839 PMC5372567

[ref10] Dong L. An M. Gu H. Y. Zhang L. G. Zhang J. B. Li C. J.. (2023). PACAP/PAC1-R activation contributes to hyperalgesia in 6-OHDA-induced Parkinson's disease model rats via promoting excitatory synaptic transmission of spinal dorsal horn neurons. Acta Pharmacol. Sin. 44, 2418–2431. doi: 10.1038/s41401-023-01141-3, PMID: 37563446 PMC10692161

[ref11] Edwards K. A. Zup S. L. (2021). Serotonin pretreatment abolishes sex-specific NMDA-induced seizure behavior in developing rats. Neuroscience 463, 184–196. doi: 10.1016/j.neuroscience.2021.03.033, PMID: 33838289

[ref12] Fathi M. Vakili K. Yaghoobpoor S. Tavasol A. Jazi K. Hajibeygi R.. (2022). Dynamic changes in metabolites of the kynurenine pathway in Alzheimer's disease, Parkinson's disease, and Huntington's disease: a systematic review and meta-analysis. Front. Immunol. 13:997240. doi: 10.3389/fimmu.2022.997240, PMID: 36263032 PMC9574226

[ref13] Fazio F. Lionetto L. Curto M. Iacovelli L. Cavallari M. Zappulla C.. (2016). Xanthurenic acid activates mGlu2/3 metabotropic glutamate receptors and is a potential trait marker for schizophrenia. Sci. Rep. 5:17799. doi: 10.1038/srep17799, PMID: 26643205 PMC4672300

[ref14] Frisardi V. Santamato A. Cheeran B. (2016). Parkinson's disease: new insights into pathophysiology and rehabilitative approaches. Parkinsons Dis. 2016, 1–2. doi: 10.1155/2016/3121727 PMC4944068 27446627

[ref15] Gerlach M. Gsell W. Kornhuber J. Jellinger K. Krieger V. Pantucek F.. (1996). A post mortem study on neurochemical markers of dopaminergic, GABA-ergic and glutamatergic neurons in basal ganglia-thalamocortical circuits in Parkinson syndrome. Brain Res. 741, 142–152. doi: 10.1016/S0006-8993(96)00915-8, PMID: 9001716

[ref16] Gu Y. Zhang J. Zhao X. Nie W. Xu X. Liu M.. (2024). Olfactory dysfunction and its related molecular mechanisms in Parkinson's disease. Neural Regen. Res. 19, 583–590. doi: 10.4103/1673-5374.380875, PMID: 37721288 PMC10581567

[ref17] Hajj R. Milet A. Toulorge D. Cholet N. Laffaire J. Foucquier J.. (2015). Combination of acamprosate and baclofen as a promising therapeutic approach for Parkinson's disease. Sci. Rep. 5:16084. doi: 10.1038/srep16084, PMID: 26542636 PMC4635348

[ref18] Heilman P. L. Wang E. W. Lewis M. M. Krzyzanowski S. Capan C. D. Burmeister A. R.. (2020). Tryptophan metabolites are associated with symptoms and Nigral pathology in Parkinson's disease. Mov. Disord. 35, 2028–2037. doi: 10.1002/mds.28202, PMID: 32710594 PMC7754343

[ref19] Holeček M. (2023). Aspartic acid in health and disease. Nutrients 15:4023. doi: 10.3390/nu15184023, PMID: 37764806 PMC10536334

[ref20] Iovino L. Tremblay M. E. Civiero L. (2020). Glutamate-induced excitotoxicity in Parkinson's disease: the role of glial cells. J. Pharmacol. Sci. 144, 151–164. doi: 10.1016/j.jphs.2020.07.011, PMID: 32807662

[ref21] Iwasaki Y. Ikeda K. Shiojima T. Kinoshita M. (1992). Increased plasma concentrations of aspartate, glutamate and glycine in Parkinson's disease. Neurosci. Lett. 145, 175–177. doi: 10.1016/0304-3940(92)90015-Y, PMID: 1361223

[ref22] Jiang X. Zhang L. Liu H. Su H. Jiang J. Qiang C.. (2023). Efficacy of non-pharmacological interventions on depressive symptoms in patients with Parkinson's disease: a study protocol for a systematic review and network meta-analysis. BMJ Open 13:e068019. doi: 10.1136/bmjopen-2022-068019, PMID: 37130665 PMC10163538

[ref23] Jing X. Yuan X. Luo X. Zhang S.-Y. Wang X.-P. (2023). An update on nondopaminergic treatments for motor and non-motor symptoms of Parkinson's disease. Curr. Neuropharmacol. 21, 1806–1826. doi: 10.2174/1570159X20666220222150811, PMID: 35193486 PMC10514518

[ref24] Kim J.-S. Kim M.-G. Ryu J. E. Lee Y.-B. Liu Q. F. Kim K. K.. (2024). Effect of woohwangchungsimwon and donepezil co-treatment on cognitive function and serum metabolic profiles in a scopolamine-induced model of Alzheimer's disease. J. Ethnopharmacol. 319:117359. doi: 10.1016/j.jep.2023.117359, PMID: 37924999

[ref25] Lei S. Zavala-Flores L. Garcia-Garcia A. Nandakumar R. Huang Y. Madayiputhiya N.. (2014). Alterations in energy/redox metabolism induced by mitochondrial and environmental toxins: a specific role for glucose-6-phosphate-dehydrogenase and the pentose phosphate pathway in paraquat toxicity. ACS Chem. Biol. 9, 2032–2048. doi: 10.1021/cb400894a, PMID: 24937102 PMC4168797

[ref26] Lemos J. C. Friend D. M. Kaplan A. R. Shin J. H. Rubinstein M. Kravitz A. V.. (2016). Enhanced GABA transmission drives bradykinesia following loss of dopamine D2 receptor signaling. Neuron 90, 824–838. doi: 10.1016/j.neuron.2016.04.040, PMID: 27196975 PMC4882167

[ref27] Li G. Ma J. Cui S. He Y. Xiao Q. Liu J.. (2019). Parkinson's disease in China: a forty-year growing track of bedside work. Transl. Neurodegener. 8:22. doi: 10.1186/s40035-019-0162-z, PMID: 31384434 PMC6668186

[ref28] Lim C. K. Fernández-Gomez F. J. Braidy N. Estrada C. Costa C. Costa S.. (2017). Involvement of the kynurenine pathway in the pathogenesis of Parkinson's disease. Prog. Neurobiol. 155, 76–95. doi: 10.1016/j.pneurobio.2015.12.009, PMID: 27072742

[ref29] Liu X.-Y. Wang K. Deng X.-H. Wei Y.-H. Guo R. Liu S.-F.. (2023). Amelioration of olfactory dysfunction in a mouse model of Parkinson's disease via enhancing GABAergic signaling. Cell Biosci. 13:101. doi: 10.1186/s13578-023-01049-9, PMID: 37270503 PMC10239587

[ref30] Lovelace M. D. Varney B. Sundaram G. Lennon M. J. Lim C. K. Jacobs K.. (2017). Recent evidence for an expanded role of the kynurenine pathway of tryptophan metabolism in neurological diseases. Neuropharmacology 112, 373–388. doi: 10.1016/j.neuropharm.2016.03.024, PMID: 26995730

[ref31] Mally J. Szalai G. Stone T. W. (1997). Changes in the concentration of amino acids in serum and cerebrospinal fluid of patients with Parkinson's disease. J. Neurol. Sci. 151, 159–162. doi: 10.1016/S0022-510X(97)00119-6, PMID: 9349670

[ref32] Mor A. Tankiewicz-Kwedlo A. Krupa A. Pawlak D. (2021). Role of kynurenine pathway in oxidative stress during neurodegenerative disorders. Cells 10:1603. doi: 10.3390/cells10071603, PMID: 34206739 PMC8306609

[ref33] Morimoto R. Iijima M. Okuma Y. Suzuki K. Yoshii F. Nogawa S.. (2023). Associations between non-motor symptoms and patient characteristics in Parkinson's disease: a multicenter cross-sectional study. Front. Aging Neurosci. 15:1252596. doi: 10.3389/fnagi.2023.1252596, PMID: 37744394 PMC10511748

[ref34] Ó Breasail M. Smith M. D. Tenison E. Henderson E. J. Lithander F. E. (2022). Parkinson's disease: the nutrition perspective. Proc. Nutr. Soc. 81, 12–26. doi: 10.1017/S0029665121003645, PMID: 35105409

[ref35] Obeso J. A. Rodríguez-Oroz M. C. Benitez-Temino B. Blesa F. J. Guridi J. Marin C.. (2008). Functional organization of the basal ganglia: therapeutic implications for Parkinson's disease. Mov. Disord. 23, S548–S559. doi: 10.1002/mds.22062, PMID: 18781672

[ref36] Öz G. Terpstra M. Tkáč I. Aia P. Lowary J. Tuite P. J.. (2006). Proton MRS of the unilateral substantia nigra in the human brain at 4 tesla: detection of high GABA concentrations. Magn. Reson. Med. 55, 296–301. doi: 10.1002/mrm.20761, PMID: 16408282

[ref37] Pasquini J. Ceravolo R. Qamhawi Z. Lee J.-Y. Deuschl G. Brooks D. J.. (2018). Progression of tremor in early stages of Parkinson's disease: a clinical and neuroimaging study. Brain 141, 811–821. doi: 10.1093/brain/awx376, PMID: 29365117

[ref38] Platten M. Nollen E. A. A. Röhrig U. F. Fallarino F. Opitz C. A. (2019). Tryptophan metabolism as a common therapeutic target in cancer, neurodegeneration and beyond. Nat. Rev. Drug Discov. 18, 379–401. doi: 10.1038/s41573-019-0016-5, PMID: 30760888

[ref39] Pontone G. M. Bakker C. C. Chen S. Mari Z. Marsh L. Rabins P. V.. (2016). The longitudinal impact of depression on disability in Parkinson disease. Int. J. Geriatr. Psychiatry 31, 458–465. doi: 10.1002/gps.4350, PMID: 26284815 PMC6445642

[ref40] Qin W. Shi Y. Chen W. Jia X. Asakawa T. (2022). Can kynurenine pathway be considered as a next-generation therapeutic target for Parkinson's disease? An update information. Biosci. Trends 16, 249–256. doi: 10.5582/bst.2022.01352, PMID: 36002303

[ref41] Samizadeh M.-A. Fallah H. Toomarisahzabi M. Rezaei F. Rahimi-Danesh M. Akhondzadeh S.. (2023). Parkinson's disease: a narrative review on potential molecular mechanisms of sleep disturbances, REM behavior disorder, and melatonin. Brain Sci. 13:914. doi: 10.3390/brainsci13060914, PMID: 37371392 PMC10296587

[ref42] Sarkar S. Raymick J. Imam S. (2016). Neuroprotective and therapeutic strategies against Parkinson's disease: recent perspectives. Int. J. Mol. Sci. 17:904. doi: 10.3390/ijms17060904, PMID: 27338353 PMC4926438

[ref43] Shao Y. Li T. Liu Z. Wang X. Xu X. Li S.. (2021). Comprehensive metabolic profiling of Parkinson's disease by liquid chromatography-mass spectrometry. Mol. Neurodegener. 16:4. doi: 10.1186/s13024-021-00425-8, PMID: 33485385 PMC7825156

[ref44] Sorgdrager F. J. H. Vermeiren Y. van Faassen M. van der Ley C. Nollen E. A. A. Kema I. P.. (2019). Age- and disease-specific changes of the kynurenine pathway in Parkinson's and Alzheimer's disease. J. Neurochem. 151, 656–668. doi: 10.1111/jnc.14843, PMID: 31376341 PMC6899862

[ref45] Stocchi F. Angelo Antonini Barone P. Bellelli G. Fagiolini A. Ferini Strambi L.. (2023). Exploring depression in Parkinson's disease: an Italian Delphi consensus on phenomenology, diagnosis, and management. Neurol. Sci. 44, 3123–3131. doi: 10.1007/s10072-023-06740-w, PMID: 37100925 PMC10415449

[ref46] Titova N. Chaudhuri K. R. (2018). Non-motor Parkinson disease: new concepts and personalised management. Med. J. Aust. 208, 404–409. doi: 10.5694/mja17.00993, PMID: 29764353

[ref47] Wang X.-L. Feng S.-T. Wang Y.-T. Chen B. Wang Z.-Z. Chen N.-H.. (2022). Comparative efficacy and acceptability of drug treatments for Parkinson's disease with depression: a systematic review with network meta-analysis. Eur. J. Pharmacol. 927:175070. doi: 10.1016/j.ejphar.2022.175070, PMID: 35659968

[ref48] Warepam M. Mishra A. K. Sharma G. S. Kumari K. Krishna S. Khan M. S. A.. (2021). Brain metabolite, N-Acetylaspartate is a potent protein aggregation inhibitor. Front. Cell. Neurosci. 15:617308. doi: 10.3389/fncel.2021.617308, PMID: 33613199 PMC7894078

[ref49] Wile D. J. Agarwal P. A. Schulzer M. Mak E. Dinelle K. Shahinfard E.. (2017). Serotonin and dopamine transporter PET changes in the premotor phase of LRRK2 parkinsonism: cross-sectional studies. Lancet Neurol. 16, 351–359. doi: 10.1016/S1474-4422(17)30056-X, PMID: 28336296 PMC5477770

[ref50] Wilson H. Dervenoulas G. Pagano G. Koros C. Yousaf T. Picillo M.. (2019). Serotonergic pathology and disease burden in the premotor and motor phase of A53T α-synuclein parkinsonism: a cross-sectional study. Lancet Neurol. 18, 748–759. doi: 10.1016/S1474-4422(19)30140-1, PMID: 31229470

[ref51] Yuan Y.-S. Zhou X.-J. Tong Q. Zhang L. Zhang L. Qi Z.-Q.. (2013). Change in plasma levels of amino acid neurotransmitters and its correlation with clinical heterogeneity in early Parkinson's disease patients. CNS Neurosci. Ther. 19, 889–896. doi: 10.1111/cns.12165, PMID: 23981689 PMC6493594

[ref52] Zárate R. V. Hidalgo S. Navarro N. Molina-Mateo D. Arancibia D. Rojo-Cortés F.. (2022). An early disturbance in serotonergic neurotransmission contributes to the onset of parkinsonian phenotypes in *Drosophila melanogaster*. Cells 11:1544. doi: 10.3390/cells11091544, PMID: 35563850 PMC9105628

[ref53] Zhang S. Collier M. E. W. Heyes D. J. Giorgini F. Scrutton N. S. (2021). Advantages of brain penetrating inhibitors of kynurenine-3-monooxygenase for treatment of neurodegenerative diseases. Arch. Biochem. Biophys. 697:108702. doi: 10.1016/j.abb.2020.108702, PMID: 33275878 PMC8111166

[ref54] Zhang L. Zhang L. Dong J. Zhao Y. Wang X.-P. (2022). Factors contributing to malnutrition in Parkinson's disease patients with freezing of gait. Front. Neurol. 13:816315. doi: 10.3389/fneur.2022.816315, PMID: 35359625 PMC8963416

[ref55] Zhang Z. Zhang S. Fu P. Zhang Z. Lin K. Ko J. K. S.. (2019). Roles of glutamate receptors in Parkinson's disease. Int. J. Mol. Sci. 20:4391. doi: 10.3390/ijms20184391, PMID: 31500132 PMC6769661

[ref56] Zhu Y. H. Huan F. Wang J. F. Xie X. Yu G. Wang X.. (2020). 1-Methyl-4-phenyl-1,2,3,6-tetrahydropyridine induced Parkinson's disease in mouse: potential association between neurotransmitter disturbance and gut microbiota Dysbiosis. ACS Chem. Neurosci. 11, 3366–3376. doi: 10.1021/acschemneuro.0c00475, PMID: 32926778

