# Supplementary material for: Serum neurotransmitter analysis of motor and non-motor symptoms in Parkinson’s patients
Source: Front Aging Neurosci. 2024 Nov 25;16:1423120. doi: 10.3389/fnagi.2024.1423120 (PMC11625801; doi:10.3389/fnagi.2024.1423120)
Supplement: Supplementary file 1 [file Table_1.DOCX]

**Table S1.** Clinical baseline data for Parkinson's patients and controls

| Group | UPDRS score | NMSS score | MMSE  score | HAMA score | HAMD score | Gender | Family history |
| --- | --- | --- | --- | --- | --- | --- | --- |
| PD1 | - | - | - | - | - | Female | No |
| PD2 | - | - | - | - | - | Female | No |
| PD3 | 29 | 16 | 28 | 1 | 0 | Male | No |
| PD4 | 14 | 51 | 25 | 22 | 15 | Female | No |
| PD5 | 30 | 68 | 24 | 17 | 7 | Male | No |
| PD6 | 60 | 56 | 17 | 15 | 16 | Female | No |
| PD7 | 16 | 20 | 26 | 2 | 4 | Male | No |
| PD8 | 12 | 67 | 28 | 21 | 10 | Female | No |
| PD9 | 25 | 28 | 26 | 22 | 17 | Female | No |
| PD10 | 19 | 62 | - | 25 | 24 | Female | No |
| PD11 | 19 | 21 | 24 | 6 | 2 | Male | No |
| PD12 | 15 | 56 | 21 | 20 | 20 | Female | No |
| PD13 | 4 | 21 | 24 | 4 | 1 | Female | No |
| PD14 | 42 | 55 | 14 | 18 | 11 | Female | No |
| PD15 | 7 | 40 | 30 | 10 | 5 | Male | No |
| PD16 | 9 | 10 | 30 | 1 | 0 | Male | No |
| PD17 | 19 | 56 | 22 | 15 | 15 | Male | No |
| PD18 | 19 | 28 | 27 | 7 | 8 | Female | No |
| PD19 | 27 | 12 | 23 | 5 | 2 | Male | No |
| PD20 | 25 | 30 | 20 | 5 | 2 | Female | No |
| PD21 | 42 | 54 | 27 | 19 | 16 | Female | No |
| PD22 | 15 | 25 | 26 | 10 | 5 | Female | No |
| PD23 | 8 | 28 | 28 | 7 | 4 | Male | No |
| PD24 | 21 | 63 | 17 | 28 | 7 | Female | No |
| PD25 | 12 | 58 | 24 | 10 | 4 | Male | No |
| PD26 | 18 | 35 | 18 | 6 | 2 | Female | No |
| PD27 | 23 | 29 | - | 9 | 6 | Female | No |
| C1 | - | - | - | - | - | Male | No |
| C2 | - | - | - | - | - | Male | No |
| C3 | - | - | - | - | - | Female | No |
| C4 | - | - | - | - | - | Male | No |
| C5 | - | - | - | - | - | Female | No |
| C6 | - | - | - | - | - | Male | No |
| C7 | - | - | - | - | - | Male | No |
| C8 | - | - | - | - | - | Female | No |
| C9 | - | - | - | - | - | Male | No |
| C10 | - | - | - | - | - | Male | No |
| C11 | - | - | - | - | - | Female | No |
| C12 | - | - | - | - | - | Female | No |
| C13 | - | - | - | - | - | Female | No |

| Group | UPDRS score | NMSS score | MMSE  score | HAMA score | HAMD score | Gender | Family history |
| --- | --- | --- | --- | --- | --- | --- | --- |
| C14 | - | - | - | - | - | Male | No |
| C15 | - | - | - | - | - | Male | No |
| C16 | - | - | - | - | - | Male | No |
| C17 | - | - | - | - | - | Female | No |
| C18 | - | - | - | - | - | Female | No |
| C19 | - | - | - | - | - | Female | No |

ps: UPDRS: MDS Unified-Parkinson Disease Rating Scale; NMSS: Non-Motor Symptoms Scale; MMSE: Mini-Mental State Examination; HAMA: Hamilton Anxiety Scale; HAMD: Hamilton Depression Scale; PD: Parkinson's patients; C:Control
